# A hot-emitter transistor based on stimulated emission of heated carriers

**DOI:** 10.1038/s41586-024-07785-3

**Published:** 2024-08-14

**Authors:** Chi Liu, Xin-Zhe Wang, Cong Shen, Lai-Peng Ma, Xu-Qi Yang, Yue Kong, Wei Ma, Yan Liang, Shun Feng, Xiao-Yue Wang, Yu-Ning Wei, Xi Zhu, Bo Li, Chang-Ze Li, Shi-Chao Dong, Li-Ning Zhang, Wen-Cai Ren, Dong-Ming Sun, Hui-Ming Cheng

**Affiliations:** 1 Shenyang National Laboratory for Materials Science, Institute of Metal Research, Chinese Academy of Sciences, Shenyang, China; 2 School of Materials Science and Engineering, University of Science and Technology of China, Shenyang, China; 3 School of Electronic and Computer Engineering, Peking University, Shenzhen, China; 4 Institute of Technology for Carbon Neutrality, Shenzhen Institute of Advanced Technology, Chinese Academy of Sciences, Shenzhen, People’s Republic of China

**Keywords:** Electrical and electronic engineering, Electronic devices, Electronic properties and devices, Electronic properties and devices, Electronic devices

## Abstract

Hot-carrier transistors are a class of devices that leverage the excess kinetic energy of carriers. Unlike regular transistors, which rely on steady-state carrier transport, hot-carrier transistors modulate carriers to high-energy states, resulting in enhanced device speed and functionality. These characteristics are essential for applications that demand rapid switching and high-frequency operations, such as advanced telecommunications and cutting-edge computing technologies^1–5^. However, the traditional mechanisms of hot-carrier generation are either carrier injection^6–11^ or acceleration^12,13^, which limit device performance in terms of power consumption and negative differential resistance^14–17^. Mixed-dimensional devices, which combine bulk and low-dimensional materials, can offer different mechanisms for hot-carrier generation by leveraging the diverse potential barriers formed by energy-band combinations^18–21^. Here we report a hot-emitter transistor based on double mixed-dimensional graphene/germanium Schottky junctions that uses stimulated emission of heated carriers to achieve a subthreshold swing lower than 1 millivolt per decade beyond the Boltzmann limit and a negative differential resistance with a peak-to-valley current ratio greater than 100 at room temperature. Multi-valued logic with a high inverter gain and reconfigurable logic states are further demonstrated. This work reports a multifunctional hot-emitter transistor with significant potential for low-power and negative-differential-resistance applications, marking a promising advancement for the post-Moore era.

## Abstract (web summary)

A mixed-dimensional hot-emitter transistor based on mixed-dimensional graphene/germanium Schottky junctions uses stimulated emission of heated carriers, achieving an ultralow subthreshold swing and a high negative differential resistance.

## Main

Transistors can be divided into three groups according to Ng and Sze^1–3^: field-effect transistors, potential-effect transistors and hot-carrier transistors. The first two groups are represented by the metal–oxide–semiconductor field-effect transistor (MOSFET) and the bipolar junction transistor (BJT), respectively, which have achieved great success in modern integrated circuits, whereas the third group has advantages in speed and multifunction based on the excess kinetic energy of hot carriers^4,5^, mainly including the hot-electron transistor (HET) and the real-space-transfer transistor (RSTT). A HET uses a metal or a semiconductor as the base, and when electrons are injected into the base from the emitter, they become hot and fast because their energy is higher than those in the base, producing a short base transit time and a high-speed device^6,7^. Two-dimensional materials such as graphene and molybdenum disulfide (MoS_2_) have been used as the base to further reduce the base transit time because of their low atomic thickness, providing a potential terahertz operation, which is promising in the next 6G technologies^8–11^. In contrast, an RSTT uses an electrical field to accelerate carriers, which, when they become hot enough, will transfer from one route to another, resulting in high-speed operation and a negative differential resistance (NDR)^12,13^, which is highly needed in various fields such as high-frequency oscillators^14,15^.

HETs and RSTTs provide potential high performance. However, the mechanisms of the hot-carrier generation are either carrier injection or acceleration, which may limit the device performance and function^7–9,13^. Neither of these devices can provide an ultralow subthreshold swing less than 60 mV dec^−1^ beyond the Boltzmann limit, which is highly needed for modern low-power applications^16,17^. In addition, for an RSTT, the NDR is limited when the device is fabricated using silicon (Si) and germanium (Ge) technology, which is compatible with mainstream semiconductor production. A novel mechanism of hot-carrier generation is needed to improve the power consumption and NDR of hot-carrier devices.

Mixed-dimensional electronic devices fabricated by combining bulk and low-dimensional materials can utilize the advantages of different dimensional materials in terms of geometric scale, and electrical and optical performance^18–21^, and may combine these advantages to provide a novel mechanism of hot-carrier generation. For low-dimensional materials such as graphene and carbon nanotubes, carrier mobility is high, which can be used to heat carriers using an electrical field. Meanwhile, various potential barriers can be formed using different energy-band combinations of bulk and low-dimensional materials, which can be used to emit high-energy carriers. Here we report a mixed-dimensional hot-emitter transistor (HOET) based on double graphene/germanium Schottky junctions. Using stimulated emission of heated carriers, the transistor achieves a subthreshold swing lower than 1 mV dec^−1^ and an NDR with a peak-to-valley current ratio greater than 100 at room temperature. Multi-valued logic applications with a high inverter gain and reconfigurable logic states are further demonstrated based on these characteristics.

## Device structure and characteristics

**Fig. 1:**
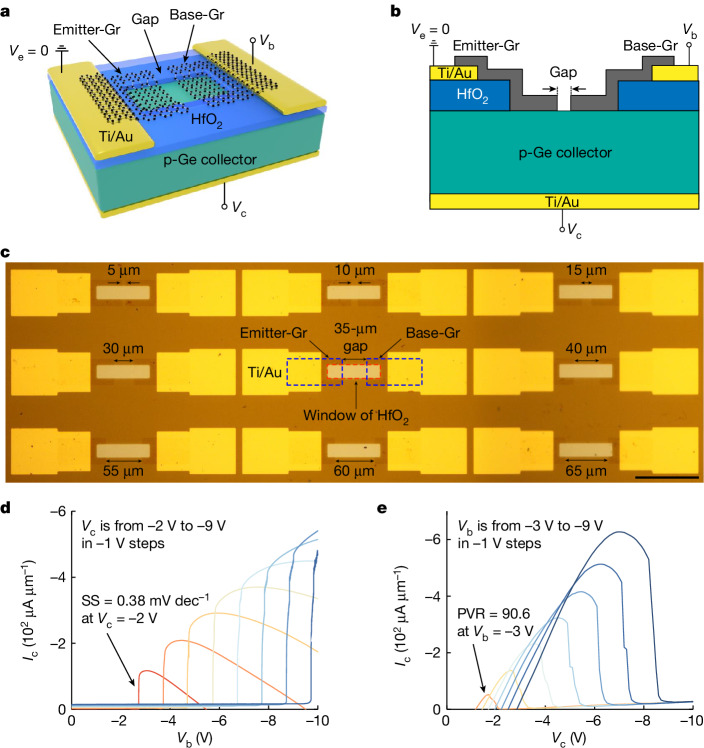
Device structure and basic characteristics. **a**, Illustration of the transistor structure, where monolayer Gr with a gap cut in it is on a p-Ge substrate (resistivity 1–10 Ω cm). The two separate Gr layers were used as the emitter (e) and base (b) contacting the Ge substrate through the HfO_2_ window, and the Ge substrate as the collector (c), with the electrodes being Ti/Au. The channel length and width of the transistor are defined as those of the HfO_2_ window. **b**, A diagram of the cross-section of the transistor showing monolayer Gr with a gap on a Ge substrate. **c**, Transistors with gap lengths of 2 μm to 75 μm were fabricated using photolithography and 9 of them are shown in the optical image. Scale bar, 100 μm. **d**, Transfer characteristics *I*_c_–*V*_b_ show a negative abruptly changing collector current *I*_c_ with an SS less than 1 mV dec^−1^ beyond the Boltzmann limit at room temperature. **e**, Output characteristics *I*_c_–*V*_c_ show an NDR with a PVR of around 100.

**Extended Data Fig. 1:**
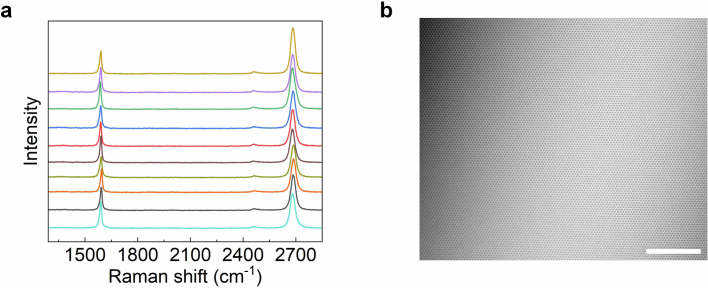
Characterization of the monolayer graphene film. **a** Raman spectra of 10 randomly selected points on the graphene film transferred onto the SiO_2_/Si substrate. The characteristic peaks observed at about 1590 cm^−1^ (G band) and about 2680 cm^−1^ (2D band) with the I_2D_ > I_G_ and the absence of defect-induced D band (1350 cm^−1^) indicate that the film is a high-quality monolayer graphene. **b** Large-area HRTEM image of the graphene film (scale bar: 5 nm). The well-defined hexagonal lattice in the absence of defects and impurities confirms the high-quality of the monolayer graphene film.

The transistor is essentially composed of a monolayer graphene (Gr) with a gap cut in it and a p-type Ge substrate. Gr contacts Ge through the hafnium dioxide (HfO_2_) window. The two separated Gr layers were used as the emitter (emitter-Gr) and the base (base-Gr), and the Ge substrate was used as the collector (Fig. 1a,b). Devices were fabricated using Gr transfer and standard semiconductor processing. The high-quality monolayer Gr was grown by chemical vapour deposition and transferred onto the Ge substrate^22^ (Extended Data Fig. 1). The gap in the Gr was fabricated using photolithography with a gap length from 2 μm to 75 μm (Fig. 1c and Methods).

**Extended Data Fig. 2:**
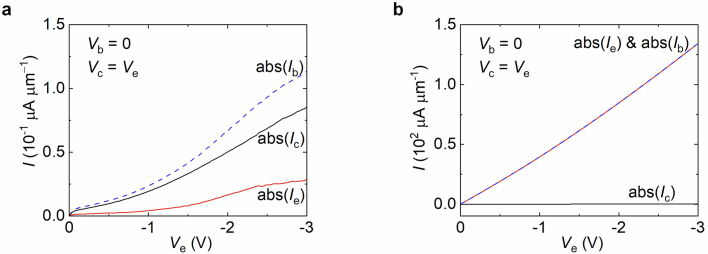
*I*−*V* characteristics of transistors with biases of *V*_b_ = 0 and *V*_c_ = *V*_e_. The devices use **a** a graphene channel with a gap and **b** a continuous graphene channel. When there is a gap in the graphene channel, the limited *I*_e_ indicates the separation of the emitter-Gr and the base-Gr. On the other hand, when a continuous graphene channel is used, the large currents (*I*_e_ = *I*_b_) are the currents in the continuous graphene channel.

**Extended Data Fig. 3:**
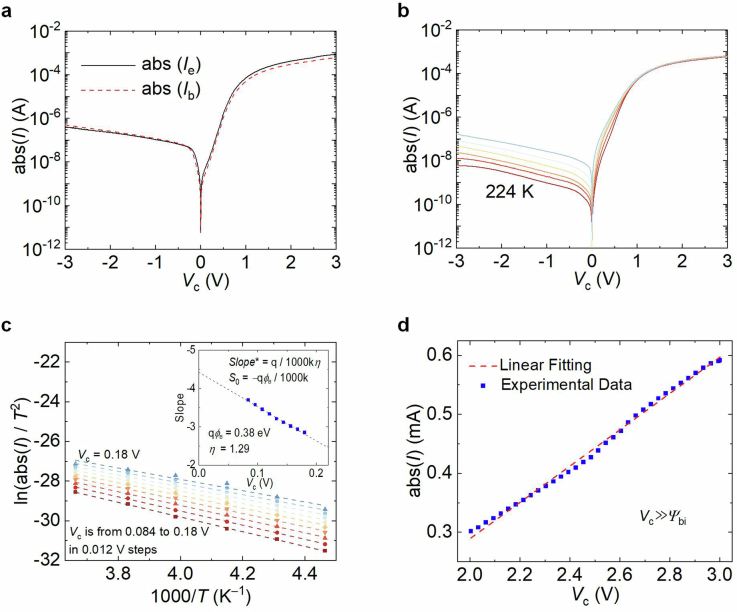
*I*−*V* characteristics of the Gr/p-Ge junctions. **a** The rectifying current ratio of about 10^3^ at ±3 V indicates the existence of a Schottky potential barrier between graphene and Ge at room temperature. Gr was grounded and *V*_c_ was applied on the Ge substrate. **b** *I*−*V* characteristics of the Gr-Ge junction measured at various temperatures from 224 K. **c** Arrhenius plots with *V*_c_ from 0.084 to 0.18 V in steps of 0.012 V. Inset: The fitted slope plotted against *V*_c_, where the y-intercept at 0 V (*S*_0_) and the slope (Slope^*^) gives a Schottky potential barrier height *qϕ*_*B*_ of 0.38 eV and an ideality factor *η* of 1.29 (Methods). **d** A series resistance of the junction of about 3 kΩ is extracted by linear fitting of the forward *I*−*V* characteristics when *V*_c_ is much larger than the built-in potential barrier height *ψ*_bi_.

The Gr is separated by the gap as tested by current–voltage (*I*–*V*) measurements (Extended Data Fig. 2). The *I*–*V* characteristics of the emitter-Gr/p-Ge junction and the base-Gr/p-Ge junction show an on-to-off current ratio of about 10^3^ at ±3 V, indicating the existence of a Schottky barrier between Gr and Ge (Extended Data Fig. 3a). The temperature-dependent *I*–*V* characteristics of the junction indicate a thermionic-emission-dominant mechanism where the Schottky barrier height was determined to be about 0.38 eV (Extended Data Fig. 3b and Methods). See Extended Data Fig. 3 for more junction characteristics. For the transistor, the relationship of the collector current *I*_c _and the base voltage *V*_b_ in the transfer characteristics (*I*_c_–*V*_b_) shows an abrupt current change beyond the Boltzmann limit where the subthreshold swing (SS) is below 1 mV dec^−1^ (Fig. 1d), whereas the one of *I*_c_ and the collector voltage *V*_c_ in the output characteristics (*I*_c_–*V*_c_) shows an NDR with a peak-to-valley current ratio (PVR) around 100 (Fig. 1e).

## Ultralow subthreshold swing

**Fig. 2:**
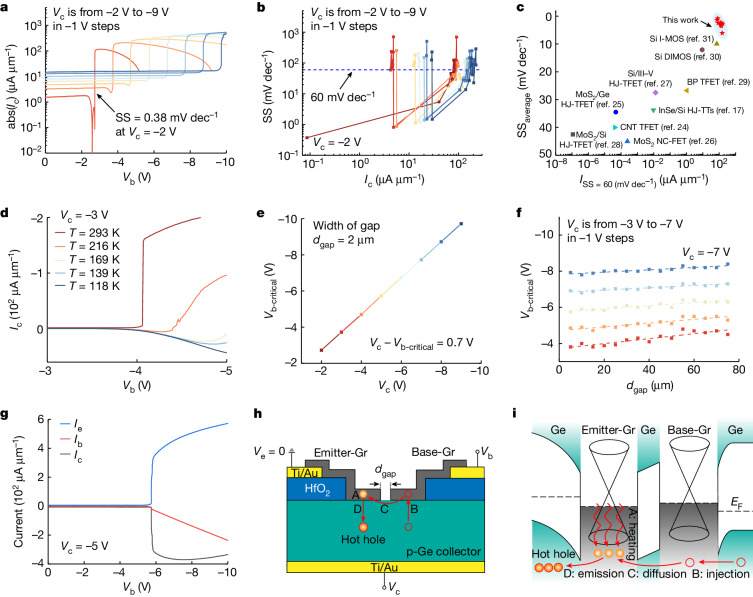
An ultralow SS and the SEHC mechanism. **a**, Transfer characteristics *I*_c_–*V*_b_ of a transistor with a 2-μm gap showing an abruptly changing collector current *I*_c_ with a minimum SS of 0.38 mV dec^−1^. **b**, SS–*I*_c_ relationship showing minimum values of SS from 0.38 mV dec^−1^ to 1.52 mV dec^−1^ and the range of the current with an SS less than 60 mV dec^−1^ of about 1 to 3 orders of magnitude. **c**, Benchmark of the average SS and maximum on-current of the transistor for a current with an SS less than 60 mV dec^−1^, which is one of the best reported results when compared with impact ionization MOSFETs (I-MOS), depletion-IMOS (DIMOS), heterojunction tunnel field-effect transistors (HJ-TFET), black phosphorus tunnel field-effect transistors (BP TFET), heterojunction tunnel triodes (HJ-TTs), carbon nanotube tunnel field-effect transistors (CNT TFET) and negative-capacitance field-effect transistors (NC-FET)^17,24–31^. **d**, Transfer characteristics with temperature-dependent current behaviour. **e**, The critical base bias *V*_b-critical_ when *I*_c_ starts to increase abruptly increases linearly with *V*_c_. **f**, For different biases of *V*_c_, devices show increasing *V*_b-critical_ with increasing gap length *d*_gap_ from 5 μm to 75 μm. **g**, As *I*_c_ increases abruptly, *I*_e_ also increases abruptly. **h**,**i**, An illustration of a transistor with a gap of a length of *d*_gap_ in the Gr channel (**h**) and its energy-band diagram near the Gr channel (**i**).

The SS is a basic parameter to characterize the switching performance of a transistor. A smaller SS is preferred for low-power operation; however, it is usually larger than 60 mV dec^−1^ because of the Boltzmann limit^23^. When the HOET works, the emitter bias *V*_e_ is grounded giving the transistor a common-emitter configuration. When the base bias *V*_b_ increases, at a critical base bias *V*_b-critical_, a negative collector current *I*_c_ is observed where the current change is rather abrupt (Figs. 1d and 2a). At room temperature, the abrupt current change is beyond the Boltzmann limit where the minimum SS is in the range of 0.38–1.52 mV dec^−1^ as *V*_c_ increases, and the range of the current with an SS less than 60 mV dec^−1^ is about 1 to 3 orders of magnitude, which could be further increased (Fig. 2b and Methods). For a current with an SS less than 60 mV dec^−1^, the average SS is from 0.82 mV dec^−1^ to 6.1 mV dec^−1^ and the maximum on-current is from 73.9 μA μm^−1^ to 165.2 μA μm^−1^, which is one of the best reported results^17,24–31^ (Fig. 2c).

## Stimulated emission of heated carrier mechanism

**Extended Data Fig. 4:**
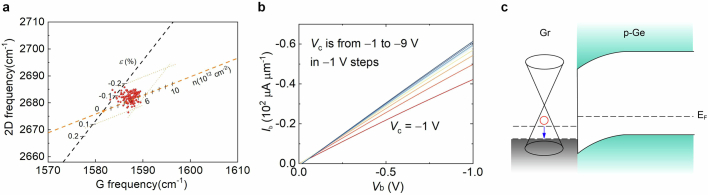
Investigation of the carrier type in the graphene channel. **a** Correlation between the frequencies of G and 2D Raman bands of the graphene on Ge, showing a typical p-type doping without a significant strain caused by processing. 130 Raman spectra were collected for the statistical analysis of doping and strain. **b** Electrical methods were used to further clarify the doping type. Input characteristics *I*_b_−*V*_b_ of a transistor with a continuous graphene channel was investigated. When the base bias *V*_b_ is less than −1 V, a collector bias *V*_c_ from −1 to −9 V leads to reverse biased emitter-Gr/Ge and base-Gr/Ge junctions. Therefore, the large *I*_b_ is the conduction current in the continuous graphene channel. When *V*_c_ increases, *I*_b_ also increases. This can be explained by **c** the corresponding energy band diagram of the Gr/Ge junction with an increasing collector bias *V*_c_. When *V*_c_ increases, because of the quantum capacitance effect of Gr, its Fermi-energy level will go down as shown using the blue arrow in the diagram. Only if the graphene is p-type so that the conduction carriers are holes, will the carrier concentration increase and lead to an increasing current. Therefore, in the HOET, the conduction carriers are holes.

**Extended Data Fig. 5:**
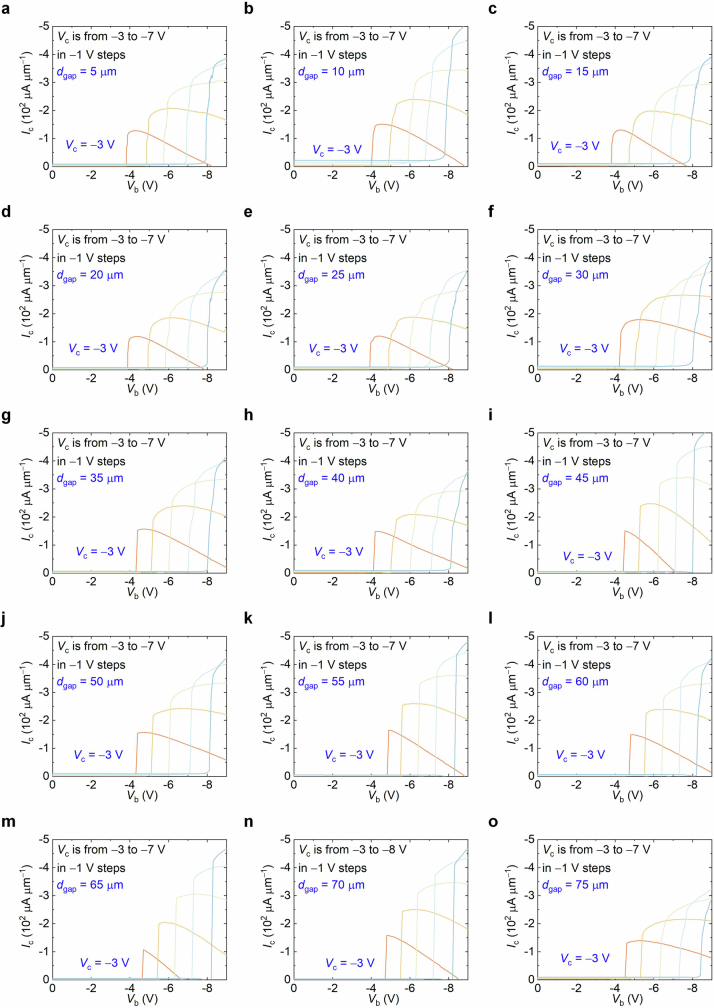
Transfer characteristics *I*_c_−*V*_b_ of HOETs. The length of gap *d*_gap_ between emitter-Gr and base-Gr increases from **a** 5 to **o** 75 μm. For each bias of *V*_c_, the critical base bias when the collector current *I*_c_ starts to increase abruptly tends to increase with *d*_gap_.

It is noted that holes are the main conduction carriers in the HOET because Gr is p-type (Extended Data Fig. 4), and the abrupt negative *I*_c_ indicates a sudden increase in the hole current flowing out of the collector, which is neither a normal reverse leakage current of the Gr/Ge junctions nor a forward current of the base-Gr/p-Ge junction. Four phenomena shed light on the device operation mechanism. First, the transfer characteristics are temperature dependent (Fig. 2d). Different from the tunnelling behaviour, the current changes more abruptly when the temperature increases. The ultralow SS appears when the temperature is above room temperature, which is the working temperature of most realistic systems. Second, the critical base bias *V*_b-critical_ when *I*_c_ abruptly changes increases linearly with *V*_c_, and *V*_c_ − *V*_b-critical_ is about 0.7 V, leading to a forward-biased base-Gr/p-Ge junction (Fig. 2e). Third, at each bias of *V*_c_, *V*_b-critical_ increases with increasing gap length *d*_gap_ (5 μm to 75 μm in 5-μm steps; Fig. 2f and Extended Data Fig. 5). Finally, *I*_c_ and *I*_e_ increase abruptly at the same time (Fig. 2g). These phenomena can be summarized as that initially both the emitter-Gr/p-Ge junction and the base-Gr/p-Ge junction are reverse biased, and when the base bias increases to a critical value, the base-Gr/p-Ge junction is sufficiently forward biased, so that an exceptional number of holes in the emitter-Gr will suddenly be emitted into the Ge collector, while holes will enter from the emitter to ensure a continuous current from the emitter to the collector. The higher the temperature, the more obvious the phenomenon, and the shorter the gap, the smaller the critical base bias.

**Extended Data Fig. 6:**
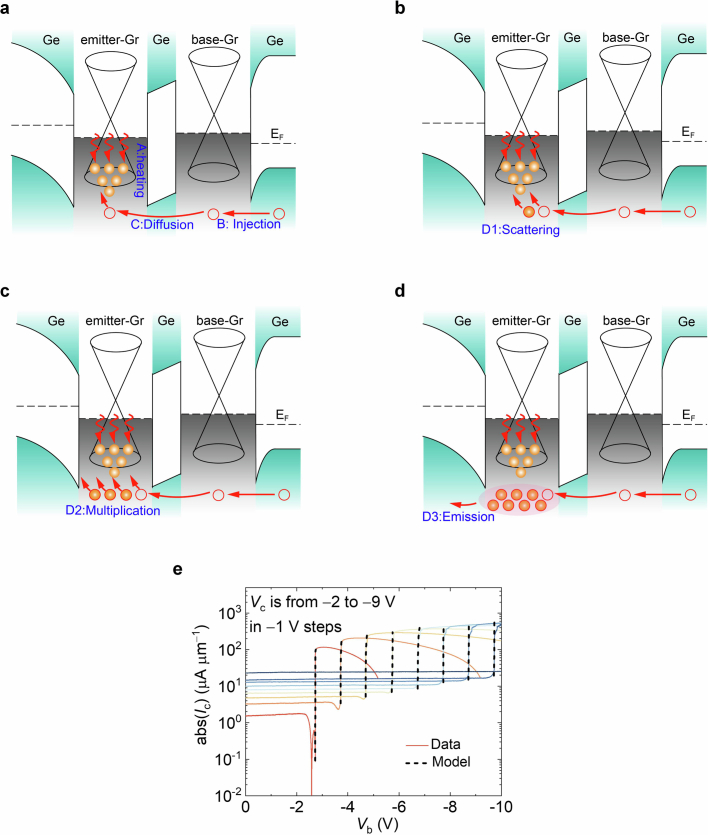
Illustrations for the emission process in the stimulated emission of heated carrier mechanism. **a** When the collector current increases abruptly, (A) the electric field in the emitter-Gr heats the holes there while (B) high-energy holes are injected from Ge into the base-Gr which will arrive at the emitter-Gr by (C) diffusion. **b** The arrived high-energy holes will pass their energy to the heated ones through (D1) carrier-carrier scattering (CCS). **c** After the stimulated holes in the emitter-Gr have enough energy from the injected holes, (D2) a multiplication process happens, before **d** the stimulated holes with enough energy are (D3) emitted into Ge with the reverse bias there, leading to the abrupt current. **e** The empirical model fits the experimental results well for the multiplication process in the HOET.

**Extended Data Fig. 7:**
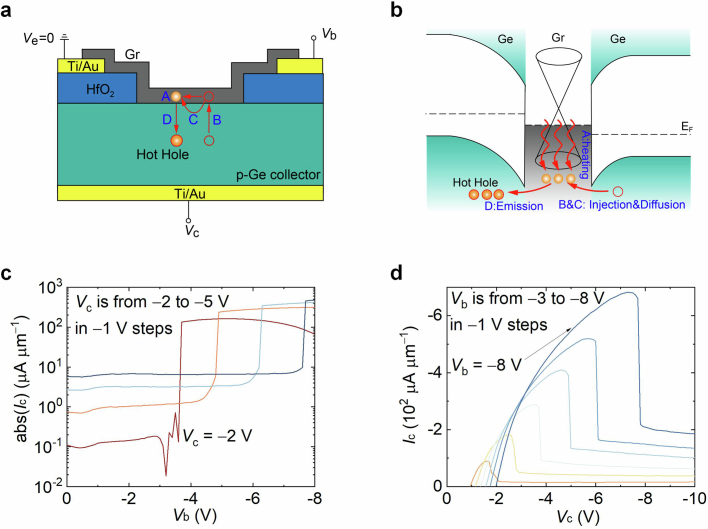
Illustrations and *I*−*V* characteristics of a transistor with a continuous graphene (Gr) channel. **a** Illustration of the device structure. **b** Energy band diagram near the Gr channel in (a). The stimulated emission of heated carrier (SEHC) mechanism indicates that even a transistor with a continuous Gr channel can generate an ultra-low SS. At a critical base bias, the left part of Gr/Ge junction will remain reverse biased while the right one will become forward biased. Four similar processes cause the ultra-low SS: A (carrier heating), B (carrier injection), C (carrier diffusion) where the injected high-energy holes will arrive at the left part of Gr by diffusion either using a path in Gr or one passing through Ge, D (carrier emission). **c** Transfer characteristics *I*_c_−*V*_b_ show a negative abruptly changing collector current *I*_c_. **d** Output characteristics *I*_c_−*V*_c_ show an obvious negative differential resistance.

We propose a stimulated emission of heated carrier (SEHC) mechanism to explain these phenomena using a structure illustration (Fig. 2h) and an energy-band diagram (Fig. 2i) of the device. There are four processes that collectively lead to the ultralow SS. In process A (carrier heating), *V*_b_ and the electric field in the emitter-Gr accelerate holes there to become heated holes; however, they are not hot enough to overcome the emitter-Gr/Ge potential barrier. In process B (carrier injection), holes are injected from Ge into the base-Gr to become high-energy holes with the forward bias there. In process C (carrier diffusion), the injected high-energy holes in the base-Gr will overcome the potential barriers induced by the base-Gr/Ge/emitter-Gr structure by diffusion to arrive at the emitter-Gr. In process D (carrier emission), with a higher energy, these arrived holes will pass their energy to the heated ones in the emitter-Gr through carrier–carrier scattering (CCS)^32^, making them the stimulated carriers that will continue to participate in the CCS process, causing a stimulated-carrier multiplication. These stimulated carriers with high energy will overcome the emitter-Gr/Ge barrier with the reverse bias there, leading to an abrupt hole current (Methods). The Ge collector current will first increase to a peak abruptly and then decrease when the current of the base-Gr/Ge junction begins to dominate. Compact modelling is used to further explain the multiplication and emission process (Methods and Extended Data Fig. 6). The SEHC mechanism indicates that even a transistor with a continuous Gr channel can still generate an ultralow SS, which is validated by our experiments (Extended Data Fig. 7).

## Negative differential resistance

**Fig. 3:**
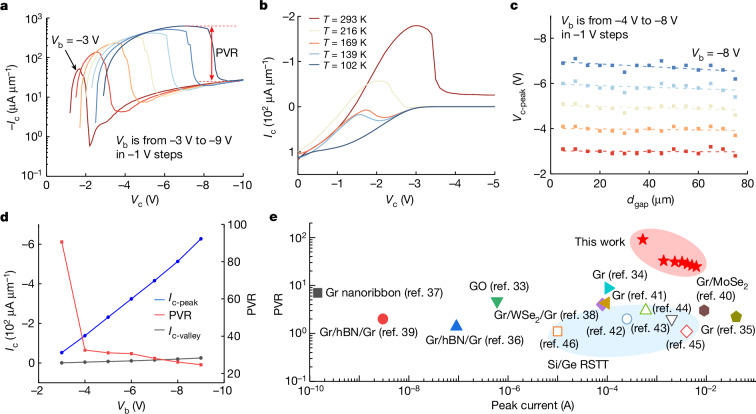
A negative differential resistance. **a**, Output characteristics *I*_c_–*V*_c_ of a transistor with a 3-μm gap showing the PVR. **b**, Temperature-dependent output characteristics. **c**, For different biases of *V*_b_, devices with an increasing gap length *d*_gap_ from 5 μm to 75 μm show a decreasing *V*_c-peak_. **d**, Peak and valley currents as well as the corresponding PVR from 90.6 to 24.6 in the output characteristics. **e**, Benchmark of the PVR for our device compared with devices using Gr and RSTTs using Si and Ge technologies^33–46^. GO, graphene oxide; hBN, hexagonal boron nitride.

**Extended Data Fig. 8:**
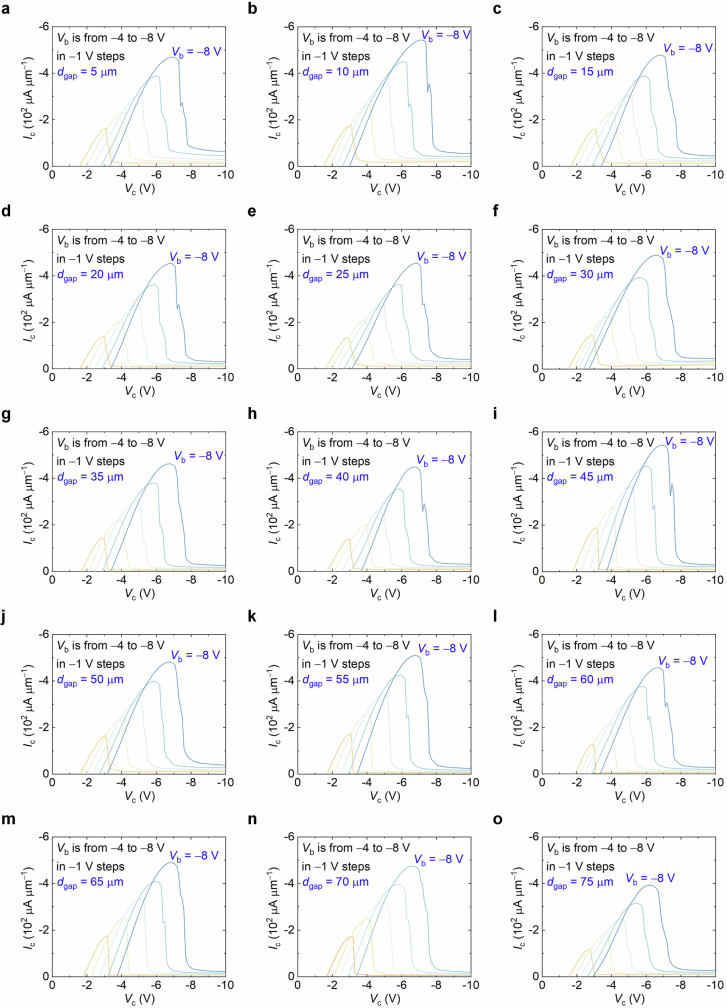
Output characteristics *I*_c_−*V*_c_ of HOETs. The length of gap *d*_gap_ between emitter-Gr and base-Gr increases from **a** 5 to **o** 75 μm. For each bias of *V*_b_, the voltage where *I*_c_ achieves its maximum tends to decrease as *d*_gap_ increases.

The NDR effect refers to the characteristic where the current of a device decreases as the voltage increases, which has been widely used in modern electronics such as amplifiers, microwave generators, high-frequency oscillators and high-speed digital-to-analogue converters, generally evaluated by the ratio of maximum and minimum currents, that is, the PVR^33–41^. However, when using Si and Ge technologies, the NDR effect generated by a hot-carrier device is limited where the PVR is no more than three^42–46^. In the HOET, the output characteristics *I*_c_–*V*_c_ show an obvious NDR (Figs. 1e and 3a). When the collector bias *V*_c_ increases, *I*_c_ first increases to a peak value, and then decreases to the reverse currents of the Gr/Ge junctions. The output characteristics are temperature dependent where the NDR gradually vanishes when the temperature decreases (Fig. 3b), and at each bias of *V*_b_, the voltage *V*_c-peak_ where *I*_c_ achieves its maximum, decreases as the gap length *d*_gap_ increases (Fig. 3c and Extended Data Fig. 8).

These phenomena are consistent with the SEHC mechanism: in the output characteristics, for each negative bias of *V*_b_, as the negative bias of *V*_c_ increases, the hot holes at the emitter-Gr are collected by the collector, leading to a large negative *I*_c_, gradually reaching the peak current. When *V*_c_ further increases, the bias of the base-Gr/Ge junction changes from a forward bias to a reverse one, and the carrier-injection process stops, leading to the valley current. It should be noted here that although an RSTT can also generate an NDR, the characteristics are different. In an RSTT, the NDR shows in the *I*_b_–*V*_b_ curve where *I*_b_ decreases as *V*_b_ increases because the carriers are accelerated by *V*_b_ to become hot and are transferred to the collector. In a HOET, *I*_b_ never decreases, no matter how large *V*_b_ is (before the device is damaged), which is also true even for a transistor with a continuous Gr channel, indicating that carriers cannot be accelerated to become hot enough only by applying *V*_b_.

**Extended Data Fig. 9:**
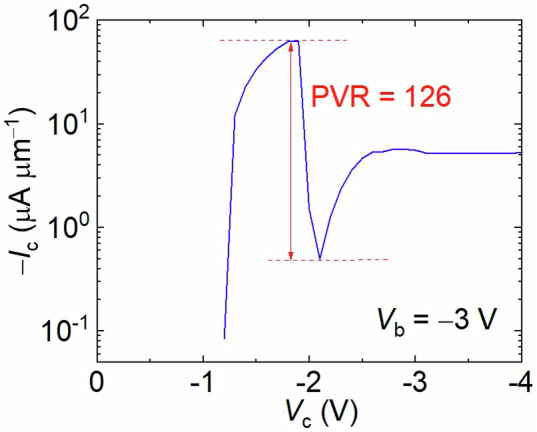
An output characteristic *I*_c_−*V*_c_ of a transistor with a 35-μm gap at *V*_b_ = −3 V. The peak to valley current ratio (PVR) is 126, higher than any Gr device and Si/Ge RSTT, and is comparable to the best two-dimensional tunnel device.

The peak and valley currents increase with the base bias *V*_b_ (Fig. 3d), and the PVR is from 90.6 to 24.6 (Fig. 3d). When *V*_b_ is −3 V, the high PVR is due to a small leakage current of the Gr/Ge junction, and the best PVR is 126 (Extended Data Fig. 9). This result is one of the highest values for a device using Gr^33–41^, and is higher than any RSTT using Si and Ge technologies^42–46^ (Fig. 3e), and is also comparable to the best result of a tunnel device using two-dimensional materials^14^.

## Multi-valued logic technology

**Fig. 4:**
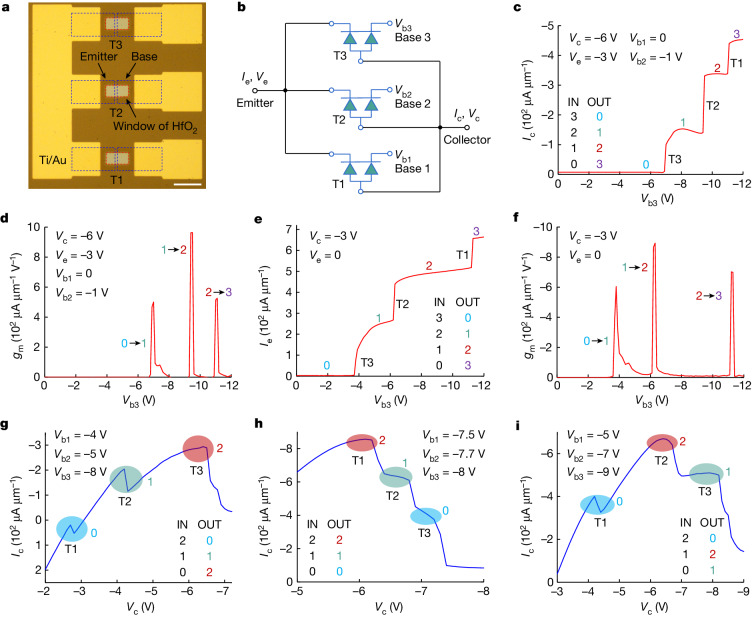
HOETs for MVL technology. **a**, In the circuit, three HOETs (T1, T2 and T3) are connected in parallel with a common Ti/Au emitter, a common Ge collector and separated bases. **b**, An equivalent circuit using device symbols showing a common emitter and collector and separated bases 1–3. **c**, The input (IN) and output (OUT) signals are voltage and current, respectively. As *V*_b3_ changes, three abrupt changes of *I*_c_ occur, showing that the circuit is a quaternary digital logic inverter. **d**, The inverter gain *g*_m_ (d*I*_c_/d*V*_b3_) can approach 1 mA μm^−1^ V^−1^ when the logic state changes. **e**,**f**, The dependence of *I*_e_ on *V*_b3_ is also the behaviour of a quaternary inverter (**e**) with a high inverter gain *g*_m_ (d*I*_e_/d*V*_b3_) (**f**). **g**–**i**, When different combinations of base biases are applied, the input logic signal *V*_c_ can be linked to different output logic signal *I*_c_, leading to a ternary digital logic inverter (**g**), a ternary follower (**h**) and a component that can be used to construct an adder (**i**).

The multifunctional HOET is promising in various applications. For example, multi-valued logic (MVL) uses more than two logic states to enable rapid and low-power data processing with high-density integration^15^, and the HOET can be used to provide a high inverter gain and reconfigurable logic states for MVL, which has rarely been reported before. A circuit was fabricated using three HOETs (T1–T3) in parallel with a common emitter, a common collector (Ge substrate) and separated bases 1–3, illustrated by an equivalent circuit and device symbols (Fig. 4a,b). The input and output signals are voltage and current, respectively^43^ where the lowest voltage potential corresponds to the input logic ‘0’ and the smallest current (absolute value) corresponds to the output logic ‘0’.

To demonstrate a high inverter gain, one base voltage is used as the input signal (IN, taking *V*_b3_ as an example), and the collector current *I*_c_ is the output signal (OUT; Fig. 4c). Other voltage biases can be applied to control the shape of the *I*_c_ curve, such as the position where an abrupt change happens. As *V*_b3_ changed from 0 V to −12 V, the injected carriers from base 3 first arrived at the emitter of device T3 leading to an abrupt change of *I*_c_, followed by another two abrupt changes when the injected carriers arrived at the emitters of devices T2 and T1. The relationship between *I*_c_ and *V*_b3_ demonstrates that the circuit can be seen as a quaternary (0, 1, 2, 3) digital logic inverter that has three characteristics. First, the inverter gain *g*_m_ (transconductance d*I*_c_/d*V*_b3_) is high when the logic state changes because of the abruptly changing *I*_c_, approaching 1 mA μm^−1^ V^−1^, which can be used to fabricate a low-power MVL (Fig. 4d). Second, more HOETs can be connected in parallel to achieve quinary or even higher system using a simple structure. Third, the dependence of *I*_e_ on *V*_b3_ is also the behaviour of a quaternary inverter, which provides more flexibility for circuit design (Fig. 4e,f).

To demonstrate reconfigurable logic states, the output characteristics *I*_c_–*V*_c_ are investigated. *V*_c_ is used as the input signal (IN), and the collector current *I*_c_ is the output signal (OUT). *I*_c_ is the sum of the collector currents of the three HOETs. For each collector current, the position where it achieves the maximum value is controlled by its base bias. Therefore, when different combinations of base biases are applied, the logic states of the output signal are different, that is, they are reconfigurable, leading to different functions. When the input logic signal is (2, 1, 0), if the output logic signal is (0, 1, 2), the circuit is a ternary digital logic inverter (Fig. 4g). If the output logic signal is (2, 1, 0), it is a ternary follower (Fig. 4h). If the output logic signal is (0, 2, 1), it can be used to construct an adder (Fig. 4i). More possibilities can be realized by using different base biases, and more HOETs can be connected in parallel to achieve higher system.

## Conclusion

Using the SEHC mechanism based on mixed-dimensional materials, the HOET provides another member of the hot-carrier transistor family, generating an ultralow SS that is one of the lowest reported values and a PVR in the NDR effect that is one of the highest for Gr devices. By combining the correct materials and device structure, the HOET can provide a multifunctional and high-performance device with potential applications in low-power and NDR technologies for the post-Moore era.

## Methods

### Preparation of the substrate

A p-type (100) Ge substrate with a resistivity of 1–10 Ω cm was cleaned by hydrofluoric acid (40 wt%) for 60 s to remove native oxide on the surface. A 30-nm-thick HfO_2_ insulating layer was deposited on top of the Ge substrate by atomic layer deposition at 200 °C (precursors, tetrakis (dimethylamido) hafnium (Hf (NMe_2_)_4_) and water). The bottom of the Ge substrate was scratched, and Ti/Au (5/50 nm) metallization was performed by electron beam evaporation to form an ohmic contact. Electrode metallization using Ti/Au (5/50 nm) on the surface was formed by photolithography and electron beam evaporation. For photolithography, photoresist s-1813 (spin-coated at 3,000 rpm for 30 s, baked at 120 °C for 2 min) and LOR3A (spin-coated at 3,000 rpm for 50 s, baked at 190 °C for 5 min) were used in sequence. The HfO_2_ layer on the substrate was then patterned by photolithography and reactive ion etching (CF_4_ 50 standard cubic centimetres per minute (sccm), 5.0 Pa, radiofrequency power 100 W, 5.5 min), followed by dilute hydrofluoric acid (5 wt%) etching for 30 s to form a window to the Ge.

### Preparation of the monolayer Gr film

Monolayer Gr film was synthesized by chemical vapour deposition on a commercial copper foil (99.9%, 25-μm thick). The copper foil was first annealed at 1,000 °C under a 5-sccm hydrogen flow and then exposed to a mixture of hydrogen (5 sccm) and methane (60 sccm) at a total pressure of 100 Pa for 30 min to grow Gr, followed by slow cooling to room temperature.

### Gr film transfer and device fabrication

Gr was transferred by using the typical wet polymethyl methacrylate (PMMA) method. The solution of PMMA (950 kDa molecular weight, Sigma, 4 wt% in ethyl lactate) was first spin-coated on the graphene/Cu foil at 2,000 rpm for 60 s and cured at 180 °C for 15 min. After removing the Cu foil by chemical etching, the PMMA/Gr film was carefully collected on the desired target substrate and baked. PMMA was then removed by immersing in acetone at 50 °C to complete the transfer. Finally, the transferred Gr was patterned by photolithography and oxygen plasma etching (200 W, 180 sccm, 2 min).

### The emission process in the SEHC mechanism

When the collector current increases abruptly, (A) the electric field in the emitter-Gr heats the holes there while (B) high-energy holes are injected from Ge into the base-Gr, which will arrive at the emitter-Gr by (C) diffusion. Only one injected hole is shown (Extended Data Fig. 6a). The arrived high-energy holes will pass their energy to the heated ones through (D1) CCS^32^ (Extended Data Fig. 6b). When the stimulated holes in the emitter-Gr gain enough energy from the injected holes, (D2) a multiplication process happens (Extended Data Fig. 6c), before the stimulated holes with enough energy are (D3) emitted into Ge with the reverse bias there, leading to the abrupt current (Extended Data Fig. 6d).

In the CCS process, after one collision between carriers, the number of carriers that can cross the Gr/Ge barrier will double, and the energy these carriers possess after the collision is still higher than the Gr/Ge barrier. In addition, the lateral electric field generated by *V*_b_ can increase these carriers’ energy. Therefore, the carriers after the collision can continue to participate in the CCS, causing the number of carriers that can cross the barrier to double again. Meanwhile, In the energy domain, the CCS will result in a high-energy-band tail in the energy distribution function, indicating an increase in high-energy carrier distribution^32^. Therefore, the continuous CCS process can cause the number of high-energy carriers in the emitter-Gr to increase repeatedly, leading to a surge in the reverse current. It should be noted that the impact ionization^47^ cannot be responsible for this phenomenon, as that *I*_c_ never increases abruptly no matter how large *V*_b_ and the corresponding electric field is applied (before the device is damaged) in the emitter-Gr if the base-Gr/Ge junction is not forward biased.

### Modelling of the CCS multiplication process

The base bias *V*_b_ determines the current *I*_c_ not only by providing a lateral electric field in the emitter-Gr but also by controlling the injected carriers at the base-Gr/Ge junction, leading to a complex relationship among the scattering, the multiplication and *V*_b_, and therefore a complex one between *I*_c_ and *V*_b_. In fact, multiplication processes are usually modelled using an experience-based approach^47^ and we provide an empirical model for the multiplication process in the HOET below.

The critical base bias *V*_b-critical_, where *I*_c_ increases abruptly, increases linearly with the collector bias *V*_c_ (Fig. 2e) and the gap length *d*_gap_ (Fig. 2f). On the basis of these experimental results, *I*_c_ is described as *I*_c_ = *M* × *I*_rev_ + *I*_0_, *M* = *A*/(1 − (*V*_b_ − *V*_c_)/(*V*_b-critical_ − *V*_c_)), *V*_b-critical_ = *Bd*_gap_ + *V*_c_ + *C*, where *I*_rev_ is the reverse current of the emitter-Gr/Ge junction before the CCS multiplication, *M* is the CCS multiplication factor and *I*_0_, *A*, *B* and *C* are fitting constants. As shown in Extended Data Fig. 6e, the model fits the experimental results well.

### Modelling of the Gr/Ge junction

On the basis of the thermionic-emission current model of a Schottky junction, the Gr/p-Ge Schottky potential barrier height *qϕ*_B_, ideality factor *η*, interface state density *D*_it_ and series resistance *R*_s_ are estimated as follows. The relationship of the forward current *I*_F_ of the Gr/Ge junction and temperature *T* is ln(*I*_F_/*T*^2^) = *C* − *q*(*ϕ*_B_ – *V*_c_/*η*)/*k* × (1/*T*), where *C* is a constant, *q* is the elementary charge, *V*_c_ is the forward voltage bias and *k* is Boltzmann’s constant^3^. Using an Arrhenius plot, the slopes of the fitted lines –*q*(*ϕ*_B_ – *V*_c_/*η*)/1,000*k* is plotted against *V*_c_ (Extended Data Fig. 3c), and the *y* intercept at 0 V is *S*_0_ = −*qϕ*_B_/1,000*k* leading to a *qϕ*_B_ of about 0.38 eV, while the slope is Slope* = *q*/1,000*kη* leading to an *η* of about 1.29. *D*_it_ is estimated using *η* and *ϕ*_B_ based on a relationship of *η* = 1 + (*δ*/*ε*_0_)(*ε*_s_/*W*_d_ + *q*^2^*D*_it_), where *δ* is the thickness of an interfacial layer between Gr and Ge, *ε*_0_ is the permittivity in vacuum (8.85 × 10^−14^ F cm^−1^), *ε*_s_ is the relative dielectric constant (16.2) of Ge and *W*_d_ is the thickness of the depletion layer of Ge (ref. ^3^). *W*_d_ = (2*ε*_s_/*qN*_a_ × (*ψ*_bi_ − *V*_c_ − *kT*/*q*))^0.5^ where *N*_a_ is the doping concentration of Ge (10^16^ cm^−3^) and *ψ*_bi_ is the built-in potential barrier height in the semiconductor as *ψ*_bi_ = *ϕ*_B_ − *ϕ*_*n*_. *ϕ*_n_ = *kT*/*q* × ln(*N*_v_/*N*_a_) where *N*_v_ is the effective states density of the valence band of Ge (5.7 × 10^18^ cm^−3^). On the basis of these models, *D*_it_ is estimated to be about 2.6 × 10^12^ cm^−2^ eV^−1^. A series resistance *R*_s_ of the junction of about 3 kΩ is extracted by a linear fitting of the forward *I*–*V* characteristic at a high voltage bias (Extended Data Fig. 3d).

### Characterization

Characterization of the graphene film and the devices was performed using a confocal Raman spectrometer (Jobin Yvon Lab RAM HR800), an optical microscope (Nikon LV100ND), a scanning electron microscope (FEI XL30 SFEG using an accelerating voltage of 10 kV) and an atomic force microscope (Bruker Dimension Icon AFM). The transistors were measured using a semiconductor analyser (Agilent B1500A with a capacitance measurement unit B1500A-A20) and a probe station (Cascade Microtech 150-PK-PROMOTION) at room temperature, and a vacuum probe station (Lake Shore TTPX/TSM1D1001) at low temperature.

For device uniformity, taking the device with a 3-μm gap as an example, 20 devices were fabricated on a wafer. The transfer characteristics (*V*_c_ = −2 V) show that for SS, sample size = 20, mean = 0.60 mV dec^−1^ and standard deviation = 0.29 mV dec^−1^, whereas the output characteristics (*V*_b_ = −4 V) show that for PVR, sample size = 20, mean = 15.80 and standard deviation = 2.84. The uniformity can be improved by advanced processes, such as using a Gr-on-Ge wafer where the Gr is grown directly on Ge instead of manual Gr transfer^48^.

### Opportunities and challenges

**Extended Data Fig. 10:**
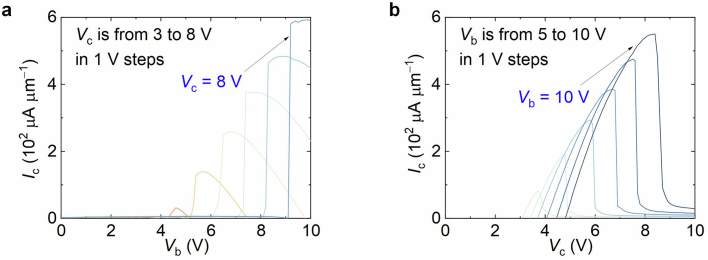
*I*−*V* characteristics of an n-type HOET fabricated based on CNT film/n-Ge junctions. A 4-μm gap in the CNT film channel was formed using photolithography. **a** Transfer characteristics *I*_c_−*V*_b_ show an abruptly changing collector current *I*_c_. Note that since electrons are the conduction carrier, the polarity of the bias is opposite to that of the p-type device. **b** Output characteristics *I*_c_−*V*_c_ show an obvious negative differential resistance. The peak-to-valley current ratio (PVR) is higher than 18.

The SEHC mechanism can be applied to devices composed of different materials. For example, using a carbon nanotube film/n-Ge junction, n-type HOETs can be fabricated (Extended Data Fig. 10). Complex circuits can be realized by using both the n-type and p-type HOETs. However, for the HOET to ultimately become practical, a series of problems must be solved including increasing the range of the current with an SS less than 60 mV dec^−1^, improving the PVR in the NDR applications and reducing the hysteresis in the characteristics.

At present, although the on-current of the transistor is high, the off-current is also high, resulting in a limited current range with a SS less than 60 mV dec^−1^, which is not an intrinsic result of the SEHC mechanism. The off-current should be reduced and the on-current increased to improve the on-to-off current ratio. To reduce the off-current, (1) the quality of the Gr/semiconductor interface should be improved by using a Gr-on-Ge wafer where the Gr is grown directly on Ge instead of being transferred^48^ and (2) other material combinations can be used, such as Gr combined with wider-bandgap semiconductors, where the potential barrier height of the Gr/semiconductor junction is higher and the intrinsic carrier concentration in the semiconductor is lower. To increase the on-current, the energy of the carriers injected from the base-Gr should be further increased, perhaps by using asymmetric potential barriers of the base/substrate and emitter/substrate junctions, which can be achieved by using different substrate semiconductor materials under the base-Gr and the emitter-Gr. These requirements are also necessary for improving the PVR in the NDR applications.

Typical transfer and output characteristics show that the width of the hysteresis window is about 0.25 V and 0.23 V, respectively. However, it should be noted that the hysteresis is not caused by the SEHC mechanism intrinsically, but by the limited quality of the Gr/Ge interface. Because of the contamination and imperfection during the transfer and fabrication process, the Gr/Ge junction itself shows a large hysteresis. This can be reduced by using a Gr-on-Ge wafer where the Gr is grown directly on Ge instead of being transferred or by using an encapsulation.

## Online content

Any methods, additional references, Nature Portfolio reporting summaries, source data, extended data, supplementary information, acknowledgements, peer review information; details of author contributions and competing interests; and statements of data and code availability are available at 10.1038/s41586-024-07785-3.

## Data Availability

Relevant data are available via Zenodo at 10.5281/zenodo.11481313 (ref. ^49^).
